# Severe Paraesophageal Hiatal Hernia Repair During a Sleeve Gastrectomy Surgery in a Morbidly Obese Patient

**DOI:** 10.7759/cureus.35897

**Published:** 2023-03-08

**Authors:** Kaveh Mozafari, Sarah Miraaj-Raza, Aadil Ilyas, Jahanvi Joshi, Vaishnavi Ganesh, Frederick Tiesenga

**Affiliations:** 1 Surgery, West Suburban Medical Center, Oak Park, USA; 2 Medicine, St. George's University School of Medicine, St. George, GRD; 3 Medicine and Surgery, Saint James School of Medicine, The Quarter, AIA; 4 Medicine and Surgery, St. George's University School of Medicine, Chicago, USA; 5 Medicine, St. George's University School of Medicine, Chicago, USA; 6 Medicine and Surgery, Saint James School of Medicine, Chicago, USA; 7 General Surgery, West Suburban Medical Center, Chicago, USA

**Keywords:** super morbid obesity, endoscopic sleeve gastrectomy, surgery general, paraesophageal hernia, paraesophageal hiatal hernia, hiatal hernia repair, sleeve gastrectomy

## Abstract

Morbid obesity increases the average risk of a patient developing a paraesophageal or hiatal hernia. Paraesophageal hernias (PEH) include several types, and their treatment is indubitably one of the most contentious topics in minimally invasive surgery. Though it is rare for PEH to manifest as a strangulated, volatilized intrathoracic stomach with infection, the increased risk of mortality is an indication for many to pursue surgical repair. Moreover, morbidly obese individuals represent a substantial rate of failure of PEH repairs. The modes of confirmation diagnostics are barium swallow or upper endoscopy. This case study focuses on a 64-year-old female who presented with several comorbidities, was appropriately evaluated for laparoscopic sleeve gastrectomy, and was previously identified to have a severe type III PEH with grade IV configuration. Additionally, the pathological finding from the extracted specimen was significant for helicobacter pylori gastritis.

## Introduction

A hernia is defined as when an internal organ pushes through a weakened area through the surrounding muscle or tissue that normally contains it. The resultant pouch that forms from the protrusion is known as the hernia and often contains different parts of the intra-abdominal organs. The severity of symptoms is mainly due to the specific organ, degree of protrusion, and area of protrusion [1]. Most hernias occur within the abdominal cavity and include inguinal, femoral, umbilical, and hiatal hernias. A hiatal hernia occurs when a portion of the stomach is displaced upward in the chest cavity through a weakened portion in the diaphragm.

Hiatal hernias can be classified as sliding hernias or paraesophageal hernias (PEH), and the distinction is primarily down to the displacement mechanism and the associated severity. Type 1 hiatus hernias possess an axial detachment in the middle of the lower esophageal sphincter (LES) and the crural diaphragm (CD) [2]. Conversely, a PEH occurs when the stomach protrudes through an open diaphragmatic hiatus alongside the esophagus. These can occur in isolation (type 2), in concurrence with an axial hiatus hernia (type 3), or with herniation of intra-abdominal organs into the thoracic cavity (type 4)[3]. Sliding hernias occur when the gastroesophageal junction (GEJ) pushes up through the hiatus. As a result, the GEJ moves up and down through the hiatus. However, a PEH occurs mainly due to abdominal organs squeezing through its associated hiatus and into the chest. Consequently, the organ is displaced next to the esophagus, which leads to more severe consequences.

PEH have multiple causes that include but are not limited to weakened tissue, repeated bouts of vigorous coughing, engaging in physical activity that involves lifting heavy objects, or repeated episodes of retching and vomiting [4]. Other risk factors include increased age and social factors that include smoking and obesity. Symptoms include radiating chest pain not alleviated by medication, dysphagia, epigastric pain, and dyspepsia. Diagnosis is primarily by barium x-ray or endoscopy [4]. Treatment methods include laparoscopic surgery that closes the hiatus and prevents the stomach from pushing through it. Oftentimes, it involves a mesh to close the hiatus. Alternate methods include fundoplication, which involves wrapping the stomach's topmost portion around the esophagus's lower affected portion.

The patient is a 64-year-old female with a past medical history significant for morbid obesity, obstructive sleep apnea (OSA), and obesity hypoventilation syndrome (OHS). Resultant imaging showed the presence of a posterior hiatal hernia, and a decision was made to engage in laparoscopic sleeve gastrectomy with posterior hiatal hernia repair.

## Case presentation

We present a case of a 64-year-old female with a past medical history of morbid obesity with a body mass index (BMI) of 42, OSA, ​​OHS, hypertension, hyperlipidemia, diabetes mellitus type 2, and arthritis. The patient snored loudly while asleep and had excessive daytime sleepiness. She felt fatigued at times to the point she had difficulty with daily chores. After various evaluations and tests, including the employment of esophagogastroduodenoscopy (EGD), it was determined that the patient had a type III PEH with grade IV configuration. The patient complained about hiatal hernia symptoms, including acid reflux, blenching, difficulties swallowing, fatigue, and severe heartburn.

The patient was diagnosed with morbid obesity with a posterior hiatal hernia. She was scheduled for laparoscopic sleeve gastrectomy with posterior hiatal hernia repair. The lab values before surgical intervention showed high glucose (129 mg/dL), low blood urea nitrogen (7 mg/dL), high chloride (108 mg/dL), and low carbon dioxide (BUN) of 19 mmol/L. Along with elevated C-reactive protein (CRP) and erythropoietin (EPO) hemoglobin A1c (HbA1c).

**Figure 1:**
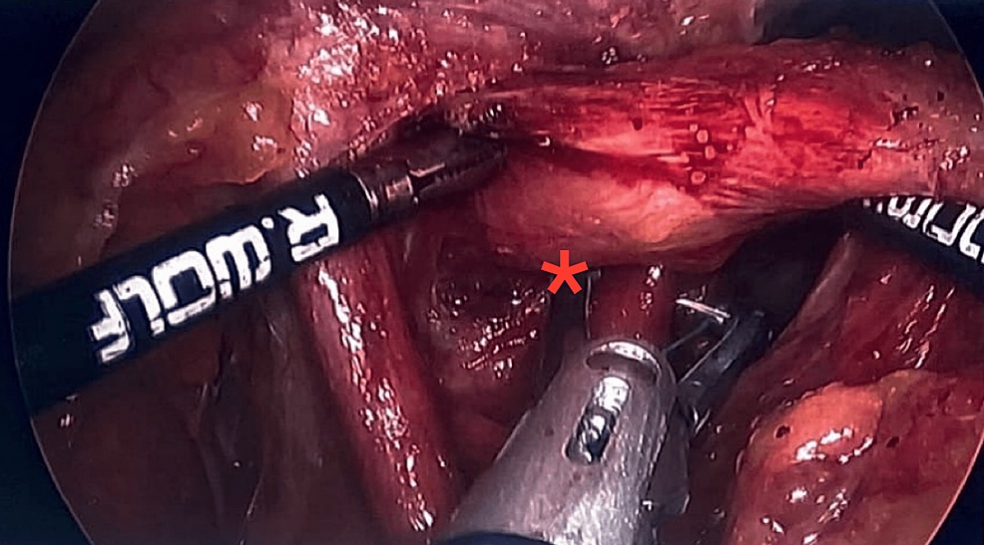
Significant hiatal hernia with advanced growth of the hernia through hiatus, the phrenoesophageal membrane stretches, displacing the GEJ past the diaphragm.

During the surgery, the patient was under general anesthesia. The abdomen was explored. The left upper quadrant and two right upper quadrant ports were placed percutaneously under direct vision as well as a liver retractor. At this time, pathology consisted of a significant hiatal hernia with advanced growth of the hernia through hiatus, the phrenoesophageal membrane stretches, displacing the GEJ past the diaphragm (Figure 1), and the stomach was then approached just lateral to the pylorus. The greater curve was taken down all the way to the angle of His. The left crus was clearly dissected out. The crural dissection was then down circumferentially, ultimately reducing the hernia. The posterior rural repair was done. Meticulous hemostasis was assured at this time. Then, the sleeve gastrectomy was performed.

A portion of the stomach was sent to the laboratory to determine any significant pathological findings. The surgical pathology report indicated the specimen measured 29.5 cm in length and was between 2.5 and 4.3 cm in diameter. The specimen was opened and revealed hemorrhagic material inside. The rugal folds were prominent. There were no exophytic lesions. Sections were placed in cassettes 1 and 2. The specimen showed Helicobacter pylori gastritis.

## Discussion

Hiatal hernias are defined as abnormal protrusion of abdominal contents into the thoracic cavity. Though there are four types of hernias, they can be broadly divided into two categories, Sliding and Paraesophageal hernias [5]. Type I or sliding hernias consist of >95% of all hiatal hernias and result from gradual laxity of the phrenoesophageal ligament or membrane. It is characterized by the protrusion of the GEJ and gastric cardia into the posterior mediastinum in the thorax, where the hernia remains intact within the phrenoesophageal membrane. However, the gastric fundus remains below the GEJ [5]. Types II, III, and IV are broadly considered PEH, though they have further distinct characterization. In type II, the gastric fundus herniates into the thoracic cavity [6]. Type III is a mix of types I and II, whereby the GEJ and fundus herniate. And type IV or complex consists of herniation of contents other than the stomach (i.e., colon, intestine, spleen) and is exceedingly rare. PEHs are considered true hernias due to a defect in the phrenoesophageal membrane and upward protrusion into the thorax of respective contents contained within a hernia sac [5].

Etiology and pathophysiology

The etiology of HH may be due to the laxity of diaphragmatic esophageal hiatus (DEH), a result of the chronic increase of intra-abdominal pressure, or even multifactorial. Laxity of DEH may be implicated in [6]. Advanced age, as the phrenoesophageal ligament weakens with age. Notably, 70% of adults who have a hiatal hernia are >70 years of age [5,6]. Smoking, as a toxin, damages elastin fibers [7]. Obesity, due to excessive deposition of fat in the hiatus, widens the circumference of DEH. Furthermore, BMI and the incidence of HH have a positive correlation [6].

Chronic increased intra-abdominal pressure may result from chronic constipation [8], pregnancy, ascites, etc. [6]. Our patient has two predisposing factors, obesity and smoking. Furthermore, though there is uncertainty as to whether it is a result of PEHs or cause, laxity of both the gastrosplenic and gastrocolic ligaments are implicated in the displacement of stomach contents [5]. If untreated, as the circumference of the hernia enlarges, there is potential for the entire stomach to roll up into the thorax, rotating around its longitudinal or transverse axis resulting in respective types of volvulus [5].

Clinical presentation

Sliding or type 1 presents with symptoms of gastroesophageal reflux disease (GERD), i.e., heartburn, regurgitation, dysphagia, and may also be asymptomatic. Complications are rare [5].

Type II, III, and IV, or PEHs, may present with: nausea, vomiting, dysphagia, postprandial fullness, i.e., early satiety, epigastric or chest pain [6]. Though GERD symptoms are less prevalent, they are a possible disposition1. PEH’s presentation corresponds with the degree of herniation and its’ impact on compressing the surrounding organs, such as the esophagus or lungs, leading to more severe symptoms. Complications are, therefore, a result of the extent of the herniation. Dysphagia can be caused by gastric volvulus, postprandial pain by gastric dissension, bleeding due to gastric volvulus, ulceration, gastritis, or erosions within the incarcerated hernia pouch. Lastly, respiratory complications may also be seen if the hernia pouch is mechanically compressing the lungs [5,6].

Diagnostics

Diagnosis of PEH is confirmed with barium swallow or upper endoscopy. Due to the herniated stomach in the thoracic cavity, a stomach bubble or retrocardiac air-fluid level may be present, suggesting PEH. Where barium swallow is a more sensitive test, an upper endoscopy aids in evaluating possible complications [5,6].

Management

The prime management of asymptotic PEH is currently controversial [9]. Some physicians recommend prophylactic surgical intervention, but most advise against the measure because there is less than a 2% yearly risk for the patients to develop complications [5]. Surgical repair is the appropriate management for symptomatic PEHs and emergent surgical repair in scenarios where the patient suffers from complications, i.e. [10] volvulus, bleeding, respiratory compromise secondary to EPH, strangulation, etc. [5,6].

## Conclusions

It can be challenging to do a sleeve gastrectomy for a patient with morbid obesity who also has a severe PEH. Although the conventional principles of hiatal hernia repair still hold, the pathophysiology seems more complicated. It necessitates a well-planned strategy based on the available data to produce the most reliable and secure long-term results. Surgical intervention is an appropriate management approach for PEH suffering from complications. Moreover, patients with indications of sleeve gastrectomy and PEH can have both procedures in one operation.
